# Interleukin-6 -174 G/C polymorphism is associated with the risk of basal cell carcinoma in a Chinese Han population

**DOI:** 10.18632/aging.103518

**Published:** 2020-08-14

**Authors:** Jing Wang, Yi Chen

**Affiliations:** 1 Department of Burn and Plastic Surgery, Huaian First People’s Hospital, Huaian, Jiangsu, China

**Keywords:** IL-6 -174 G/C polymorphism, basal cell carcinoma, case-control study

## Abstract

To explore the association between the IL-6 -174 G/C polymorphism and the risk of basal cell carcinoma (BCC) in a Chinese population, we performed a case-control study involving 265 BCC patients and 341 controls. Genotyping was performed using polymerase chain reaction with sequence-specific primers. The data showed that the GG+CG and GG genotypes were associated with an increased risk of BCC. The G allele increased the risk of BCC. Moreover, stratified analyses indicated the risk was higher in males, smokers, drinkers, and those aged ≥ 60 years. Cross-over analysis confirmed that the combined effects of the GG or CG genotype and environmental factors (smoking and alcohol consumption) contribute to an increased risk of BCC. In addition, the IL-6 -174 G/C polymorphism was related to larger tumors and multiple lesions. These findings indicate that the IL-6 -174 G/C polymorphism is associated with an increased risk of BCC in a Chinese population. This locus is thus a potential diagnostic marker for predicting susceptibility to BCC.

## INTRODUCTION

Basal cell carcinoma (BCC) is the most commonly occurring skin cancer in Caucasians [1]. Although the prognosis of BCC patients is good, the incidence of BCC is rising because of population aging and increased sun exposure [2]. BCC manifests as pearly and telangiectatic papules or nodules, with or without ulceration; or as indurated, erythematous, or ulcerated patches with a discrete papular border. Although BCC can be treated surgically, it has aggressive features that include local recurrence, tissue destruction, and widespread dissemination when BCC metastases occur [3, 4]. Exposure to ultraviolet radiation (UVR) is the most important risk factor for BCC [5]. However, not all patients with BCC have been exposed to UVR, suggesting that other, perhaps genetic, factors contribute to BCC risk [6].

Various cytokines, chemokines, and growth factors are implicated in the development of BCC [2, 7, 8]. Among the cytokines associated with the pathogenesis of BCC is interleukin (IL)-6 [9]. Overexpression of IL-6 in human BCC cell lines increases their anti-apoptotic activity and tumorigenic potency [9]. Sternberg et al. suggested that synergistic crosstalk between hedgehog and IL-6 signaling promotes BCC growth [10]. In addition, BCC tissue explants showed significantly higher IL-6 levels than squamous cell tumors [7].

Several studies have investigated the association between the IL-6 -174 G/C polymorphism and BCC risk; however, their findings were conflicting [11–13]. We therefore carried out the present case-control study to evaluate the effect of the IL-6 -174 G/C polymorphism on the risk and prognosis of BCC in a Chinese Han population.

## RESULTS

### Characteristics of the patients and controls

This case-control study enrolled 265 patients with BCC and 341 controls. Table 1 shows the demographic and clinical characteristics of the study participants. The distributions of age, sex, smoking, and alcohol consumption did not significantly differ between the two groups. Among the BCC patients, 203 (76.6%) had primary BCC and 62 (23.4%) had recurrent BCC. Multiple BCC was defined as detection of more than one tumor. The BCC patients included 215 with a history of UVR exposure and 50 without such a history.

**Table 1:** Patient demographics and risk factors in basal cell carcinoma.

**Characteristics**	**Case (N=265)**	**Control (N=341)**	***P***
Age	62.43±7.25	61.98±7.39	0.452
Sex			0.728
Male	140(52.8%)	185(54.3%)	
Female	125(47.2%)	156(45.7%)	
Smoking			0.383
Yes	126(47.5%)	150(44.0%)	
No	139(52.5%)	191(56.0%)	
Alcohol			0.578
Yes	149(56.2%)	184(54.0%)	
No	116(43.8%)	157(46.0%)	
Diagnosis			
BCC	203(76.6%)		
BCC recurrent	62(23.4%)		
Tumor size			
>1 cm	154(58.1%)		
≤1cm	111(41.9%)		
Occupational exposure to mutagens			
Yes	21(7.9%)		
No	244(92.1%)		
Number of lesions			
Single	107(40.4%)		
Multiple	158(59.6%)		
Location of lesions			
Area exposed to UV	215(81.1%)		
Area not exposed to UV	50(18.9%)		

### Relationship between the IL-6 -174 G/C polymorphism and the risk of BCC

The genotype distribution of the -174 G/C polymorphism among the controls was consistent with the Hardy-Weinberg equilibrium. The genotype and allele distributions of the IL-6 -174 G/C polymorphism differed significantly between the BCC patients and controls (Table 2). The GG and GG+CG genotypes were associated with an increased risk of BCC (GG *vs*. CC: OR, 1.89; 95% CI, 1.04–3.43; *P* = 0.037), and this association remained significant after adjusting for age and sex. Moreover, the G allele of the IL-6 -174 G/C polymorphism was related to an increased risk of BCC (G *vs*. C: OR, 1.38; 95% CI, 1.07–1.77; *P* = 0.012). Stratified analyses of sex, age, alcohol consumption, and smoking indicated that the IL-6 -174 G/C polymorphism increased the risk of BCC among males, smokers, drinkers, and those aged ≥ 60 years (Table 3).

**Table 2:** Genotype frequencies of IL-6 -174 G/C polymorphism in cases and controls.

**Models**	**Genotype**	**Case (n, %)**	**Control (n, %)**	**OR (95% CI)**	***P*-value**	*OR (95% CI)	*P-value
Co-dominant	CC	120(45.3%)	186(54.7%)	1.00(reference)	-	1.00(reference)	-
Heterozygote	CG	117(44.2%)	131(38.5%)	1.38(0.99-1.94)	0.060	1.38(0.98-1.94)	0.064
Homozygote	GG	28(10.6%)	23(6.8%)	**1.89(1.04-3.43)**	**0.037**	**1.88(1.03-3.41)**	**0.040**
Dominant	CC	120(45.3%)	186(54.7%)	1.00(reference)	-	1.00(reference)	-
	GG+CG	145(54.7%)	154(45.3%)	**1.46(1.06-2.02)**	**0.022**	**1.45(1.05-2.01)**	**0.024**
Recessive	CG+CC	237(89.4%)	317(93.2%)	1.00(reference)	-	1.00(reference)	-
	GG	28(10.6%)	23(6.8%)	1.63(0.92-2.90)	0.098	**1.62(0.91-2.89)**	**0.101**
Allele	C	357(67.4%)	503(74.0%)	1.00(reference)	-	1.00(reference)	-
	G	173(32.6%)	177(26.0%)	**1.38(1.07-1.77)**	**0.012**	**-**	**-**

The genotyping was successful in 265 cases and 340 controls for -174 G/C polymorphism;

Bold values are statistically significant (*P* < 0.05).

*Adjustment for age and sex.

**Table 3:** Stratified analyses between IL-6 -174 G/C polymorphism and the risk of basal cell carcinoma.

**Variable**	**(case/control)**			**CG vs. CC**	**GG vs. CC**	**GG vs. CC+CG**	**GG+CG vs. CC**
	**CC**	**CG**	**GG**				
Sex							
Male	67/108	55/67	18/10	1.32(0.83-2.12); 0.242	**2.90(1.26-6.66); 0.012**	**2.58(1.15-5.79); 0.021**	1.53(0.98-2.38); 0.060
Female	53/78	62/64	10/13	1.43(0.87-2.34); 0.159	1.13(0.46-2.77); 0.786	0.95(0.40-2.25); 0.907	1.38(0.86-2.21); 0.187
Smoking							
Yes	42/79	71/60	13/11	**2.23(1.34-3.70); 0.002**	2.22(0.92-5.39); 0.077	1.45(0.63-3.37); 0.383	**2.23(1.36-3.63); 0.001**
No	78/107	46/71	15/12	0.89(0.55-1.43); 0.624	1.72(0.76-3.87); 0.194	1.79(0.81-3.97); 0.148	1.01(0.65-1.57); 0.971
Alcohol							
Yes	64/100	69/76	16/7	1.42(0.90-2.23); 0.130	**3.57(1.39-9.16); 0.008**	**3.02(1.21-7.56); 0.018**	**1.60(1.04-2.47); 0.035**
No	56/86	48/55	12/16	1.34(0.80-2.24); 0.263	1.15(0.51-2.62); 0.736	1.02(0.46-2.24); 0.967	1.30(0.80-2.10); 0.288
Age (years)							
<60	49/74	44/54	8/8	1.23(0.72-2.11); 0.450	1.51(0.53-4.29); 0.439	1.38(0.50-3.80); 0.538	1.27(0.76-2.12); 0.369
≥60	71/112	73/77	20/15	1.50(0.97-2.32); 0.071	**2.10(1.01-4.38); 0.047**	1.75(0.87-3.54); 0.119	**1.60(1.05-2.41); 0.027**

Bold values are statistically significant (*P* < 0.05).

### Combined and interactive effects of the IL-6 -174 G/C polymorphism and environmental factors on BCC risk

We used cross-over analyses to assess the impact of the interactions between genetic factors and smoking or alcohol consumption on BCC risk (Table 4). For nonsmokers and nondrinkers, neither the CG nor GG genotype was more associated with BCC risk than the CC genotype. For smokers, however, carrying the CG genotype was significantly associated with an greater risk of BCC as compared to nonsmokers carrying the CC genotype (CG + smoking vs. CC + nonsmoking: OR, 1.62, 95% CI, 1.03-2.55; *P* = 0.035;). Similarly, BCC patients who drank alcohol and carried the GG or AG genotype had a higher risk of developing BCC than patients who did not drink alcohol and carried the GG genotype (GG + drinking vs. CC + non- drinking: OR, 3.51, 95% CI, 1.36-9.08; *P* = 0.007). This suggests a potential interaction between genetic and environmental factors in BCC.

**Table 4:** Genetic (G) and environmental (E) factors 2*4 fork analysis.

**G^a^**	**E^b^**	**Case**	**Control**	**OR (95%CI); P value**	**Reflecting information**
**IL-6–174G/C**					
GG vs. CC	Smoking				
+	+	13	11	1.62(0.69,3.81); 0.264	G, E combined effect
+	-	15	12	1.72(0.76,3.87); 0.190	G alone effect
-	+	42	79	0.73(0.45,1.17); 0.192	E alone effect
-	-	78	107	1.00 (reference)	Common control
CG vs. CC	Smoking				
+	+	71	60	**1.62(1.03,2.55); 0.035**	G, E combined effect
+	-	46	71	0.89(0.55,1.43); 0.624	G alone effect
-	+	42	79	0.73(0.45,1.17); 0.192	E alone effect
-	-	78	107	1.00 (reference)	Common control
GG vs. CC	Drinking				
+	+	16	7	**3.51(1.36,9.08); 0.007**	G, E combined effect
+	-	12	16	1.15(0.51,2.62); 0.736	G alone effect
-	+	64	100	0.98(0.62,1.56); 0.941	E alone effect
-	-	56	86	1.00 (reference)	Common control
CG vs. CC	Drinking				
+	+	69	76	1.39(0.87,2.23); 0.164	G, E combined effect
+	-	48	55	1.34(0.80,2.24); 0.263	G alone effect
-	+	64	100	0.98(0.62,1.56); 0.941	E alone effect
-	-	56	86	1.00 (reference)	Common control

^a^G (+): IL-6 gene 174 G/C variants (Heterozygous or homozygous); G (-): wild type

^b^E(+): smoking/non-smoking; E(-): non-smoking/non-drinking

### Correlations between the IL-6 -174 G/C polymorphism and the clinicopathological characteristics of BCC

We evaluated the association between the IL-6 -174 G/C polymorphism and the clinicopathological characteristics of patients with BCC (Table 5). We found that the IL-6 -174 G/C polymorphism was associated with BCC risk, larger tumors (> 1 cm), and multiple lesions. No association between the IL-6 -174 G/C polymorphism and BCC risk was detected in subgroup analyses of occupational exposure to mutagens and location of lesions.

**Table 5:** The associations between IL-6 -174 G/C polymorphism and clinical characteristics of basal cell carcinoma.

**Characteristics**	**Genotype distributions**			
	**CC**	**CG**	**GG**	**CG+GG**
Diagnosis				
BCC/BCC recurrent	83/37	95/22	25/3	120/25
OR (95%CI); *P*-value	1.0 (reference)	**1.93(1.05-3.52); 0.034**	**3.72(1.06-13.08); 0.041**	**2.14(1.20-3.82); 0.010**
Tumor size				
>1 cm/≤1 cm	59/61	75/42	20/8	95/50
OR (95%CI); *P*-value	1.0 (reference)	**1.85(1.10-3.11); 0.021**	**2.59(1.06-6.32); 0.037**	**1.96(1.20-3.22); 0.008**
Occupational exposure to mutagens				
Yes/No	7/113	10/107	4/24	14/131
OR (95%CI); *P*-value	1.0 (reference)	1.51(0.55-4.11); 0.421	2.69(0.73-9.92); 0.137	1.73(0.67-4.42); 0.256
Number of lesions				
Multiple/Single	37/83	55/62	15/13	70/75
OR (95%CI); *P*-value	1.0 (reference)	**1.99(1.17-3.38); 0.011**	**2.59(1.12-5.98); 0.026**	**2.09(1.26-3.47); 0.004**
Location of lesions				
Area exposed to UV/Not	100/20	94/23	21/7	115/30
OR (95%CI); *P*-value	1.0 (reference)	0.82(0.42-1.59); 0.551	0.60(0.23-1.60); 0.307	0.77(0.41-1.43); 0.406

Bold values are statistically significant (*P* < 0.05).

## DISCUSSION

In this case-control study, we found that the IL-6 -174 G/C polymorphism is associated with an increased risk of BCC in a Chinese population. Moreover, stratified analyses showed that interactions between the IL-6 -174 G/C polymorphism and sex, age, alcohol consumption, and smoking contribute to the risk of BCC. In addition, the -174 G/C polymorphism was correlated with BCC risk (but not recurrent BCC), larger tumors, and multiple lesions.

IL-6 is a pivotal link between inflammation and cancer [14]. UVR, a key risk factor for BCC, stimulates the release of cytokines, including IL-6, from skin cells [15], and IL-6 is overexpressed in BCC [7]. Jee et al. showed that overexpression of IL-6 increased anti-apoptotic activity and tumorigenic potency in human BCC cell lines [9]. In addition, Sławińska et al. reported that the serum IL-6 level is significantly higher in BCC patients than in healthy controls [13].

The IL-6 gene is located on chromosome 7p21. The IL-6 -174 G/C polymorphism may lead to higher transcriptional activity, resulting in a higher serum IL-6 levels [16]. Several studies have explored the association between the IL-6 -174 G/C polymorphism and the risk of cancer. The meta-analysis from Zhou et al. revealed a non-significant association between the IL-6 -174 G/C polymorphism and overall cancer risk [17]. On the other hand, Chen et al. found that the IL-6 -174 G/C polymorphism protected against polycystic ovary syndrome [18]. And Duan et al. reported that the -174 G/C polymorphism was a low-penetrance susceptibility variant for cervical cancer [19]. In addition, the IL-6 -174 G/C polymorphism reportedly enhances the susceptibility of African American men to prostate cancer [20].

Three prior studies investigated the relationship between the IL-6 -174 G/C polymorphism and the risk of BCC [12–14]. Zhang et al. reported that the IL-6 -174 G/C polymorphism is not associated with the risk of BCC in China [14]. Similar findings were reported in a subsequent study carried out in Sweden and Finland [12]. By contrast, Sławińska et al. reported that the IL-6 -174 G/C polymorphism is related to an increased risk of BCC in a population from northern Poland [13]. Consistent with that report, we found in the present study that the IL-6 -174 G/C polymorphism was associated with an increased risk of BCC in a Chinese Han population. The conflicting findings of these earlier studies [12–14] may reflect differences in the sample sizes, ethnicity of the subjects, clinical heterogeneity, and genotyping methods. To our knowledge, this is the first Chinese study to detect an association between the IL-6 -174 G/C polymorphism and the risk of BCC. Moreover, stratified analyses showed that the risk of BCC was significantly higher in males, smokers, drinkers, and those aged ≥ 60 years. Thus, interactions between the -174 G/C polymorphism and these factors likely contribute to the increased risk of BCC. To further evaluate the impact of interactions between environmental and genetic factors on BCC susceptibility, we performed cross-over analyses, which revealed that CG genotype carriers who were smokers or GG genotype carriers who were drinkers had poorer overall survival among BCC patients.

This study has several limitations. First, the sample size was small. Second, the genotype distribution was not representative of that in the general population, which may have resulted in selection bias. Third, only one IL-6 gene SNP was investigated; other functional IL-6 gene polymorphisms should be studied. Fourth, the impact of the IL-6 -174 G/C polymorphism on the therapeutic efficacy of imiquimod was not evaluated.

## CONCLUSION

The IL-6 -174 G/C polymorphism is associated with an increased risk of BCC. Further studies with larger sample sizes in other populations will be needed to confirm these findings.

## MATERIALS AND METHODS

### Subjects

We recruited 265 patients with BCC and 341 controls from the Affiliated Huaian No.1 People’s Hospital of Nanjing Medical University. The diagnosis of BCC was based on histopathological findings and clinical signs. The exclusion criteria were a history of cancer and other skin diseases or taking immunosuppressive drugs. Patients with genetic syndromes associated with multiple BCCs were also excluded. Individuals exposed to UVR for more than one hour/day of work outside between 12:00 and 16:00 for >60 days were defined having the history of ultraviolet irradiation. We mainly evaluated whether head, face, neck and the back of the hand were exposed to UVR. The controls were individuals who underwent health examinations at the same hospital. Informed consent was obtained from all individuals before enrollment in the study. This study was approved by the Ethics Committee of the Affiliated Huaian No.1 People’s Hospital of Nanjing Medical University and was carried out in accordance with the tenets of the Declaration of Helsinki.

### Blood sampling and genotyping

Peripheral blood (2 mL) was collected from all study participants and treated with EDTA. Genomic DNA was extracted from the peripheral blood samples using a TIANamp Blood DNA kit (Tiangen Biotech, Beijing, China). IL-6 -174 G/C polymorphism was genotyped using polymerase chain reaction with sequence-specific primers: 5’-GCCTCAATGACGACCTAAGC-3’ (forward) and 5’-GGCAGAATGAGCCTCAGACA-3’ (reverse). Approximately 10% of the BCC patients and controls were subjected to a second genotyping, and the genotypes were 100% concordant.

### Statistical analysis

The genotype of the controls was tested for compliance with Hardy-Weinberg equilibrium. The distributions of alleles and genotypes were compared between the cases and controls with the chi-squared test. Logistic regression analysis and calculation of the odds ratios (ORs) and 95% confidence intervals (CIs) were performed to assess the relationship between the IL-6 -174 G/C polymorphism and the risk of BCC. Subgroup analyses of the impacts of alcohol, smoking, sex, and age were also conducted. Logistic regression analyses were also used to evaluate the exposure combination models. Values of *P* < 0.05 were considered significant. Statistical analyses were performed using the Statistical Package for the Social Sciences (SPSS) version 22.0 (SPSS Inc., Chicago, IL, USA).

## References

[1] Russo I, Cona C, Saponeri A, Bassetto F, Baldo V, Alaibac M. Association between toll-like receptor 7 Gln11Leu single-nucleotide polymorphism and basal cell carcinoma. Biomed Rep. 2016; 4:459–62. 10.3892/br.2016.597 27073632 PMC4812152

[2] Kasper M, Jaks V, Hohl D, Toftgård R. Basal cell carcinoma - molecular biology and potential new therapies. J Clin Invest. 2012; 122:455–63. 10.1172/JCI58779 22293184 PMC3266783

[3] Lear W, Dahlke E, Murray CA. Basal cell carcinoma: review of epidemiology, pathogenesis, and associated risk factors. J Cutan Med Surg. 2007; 11:19–30. 10.2310/7750.2007.00011 17274935

[4] Rubin AI, Chen EH, Ratner D. Basal-cell carcinoma. N Engl J Med. 2005; 353:2262–69. 10.1056/NEJMra044151 16306523

[5] Nahhas AF, Scarbrough CA, Trotter S. A review of the global guidelines on surgical margins for nonmelanoma skin cancers. J Clin Aesthet Dermatol. 2017; 10:37–46. 28458773 PMC5404779

[6] Chinem VP, Miot HA. Epidemiology of basal cell carcinoma. An Bras Dermatol. 2011; 86:292–305. 10.1590/s0365-05962011000200013 21603813

[7] Elamin I, Zecević RD, Vojvodić D, Medenica L, Pavlović MD. Cytokine concentrations in basal cell carcinomas of different histological types and localization. Acta Dermatovenerol Alp Pannonica Adriat. 2008; 17:55–59. 18709290

[8] Wong DA, Bishop GA, Lowes MA, Cooke B, Barnetson RS, Halliday GM. Cytokine profiles in spontaneously regressing basal cell carcinomas. Br J Dermatol. 2000; 143:91–98. 10.1046/j.1365-2133.2000.03596.x 10886141

[9] Jee SH, Shen SC, Chiu HC, Tsai WL, Kuo ML. Overexpression of interleukin-6 in human basal cell carcinoma cell lines increases anti-apoptotic activity and tumorigenic potency. Oncogene. 2001; 20:198–208. 10.1038/sj.onc.1204076 11313947

[10] Sternberg C, Gruber W, Eberl M, Tesanovic S, Stadler M, Elmer DP, Schlederer M, Grund S, Roos S, Wolff F, Kaur S, Mangelberger D, Lehrach H, et al. Synergistic cross-talk of hedgehog and interleukin-6 signaling drives growth of basal cell carcinoma. Int J Cancer. 2018; 143:2943–54. 10.1002/ijc.31724 29987839 PMC6282712

[11] Festa F, Kumar R, Sanyal S, Undén B, Nordfors L, Lindholm B, Snellman E, Schalling M, Försti A, Hemminki K. Basal cell carcinoma and variants in genes coding for immune response, DNA repair, folate and iron metabolism. Mutat Res. 2005; 574:105–11. 10.1016/j.mrfmmm.2005.01.026 15914210

[12] Sławińska M, Zabłotna M, Gleń J, Lakomy J, Nowicki RJ, Sobjanek M. STAT3 polymorphisms and IL-6 polymorphism are associated with the risk of basal cell carcinoma in patients from northern Poland. Arch Dermatol Res. 2019; 311:697–704. 10.1007/s00403-019-01952-7 31342143 PMC6787107

[13] Zhang Z, Liu W, Jia X, Gao Y, Hemminki K, Lindholm B. Use of pyrosequencing to detect clinically relevant polymorphisms of genes in basal cell carcinoma. Clin Chim Acta. 2004; 342:137–43. 10.1016/j.cccn.2003.12.010 15026274

[14] Bromberg J, Wang TC. Inflammation and cancer: IL-6 and STAT3 complete the link. Cancer Cell. 2009; 15:79–80. 10.1016/j.ccr.2009.01.009 19185839 PMC3684978

[15] Kirnbauer R, Köck A, Neuner P, Förster E, Krutmann J, Urbanski A, Schauer E, Ansel JC, Schwarz T, Luger TA. Regulation of epidermal cell interleukin-6 production by UV light and corticosteroids. J Invest Dermatol. 1991; 96:484–89. 10.1111/1523-1747.ep12470181 2007786

[16] Fishman D, Faulds G, Jeffery R, Mohamed-Ali V, Yudkin JS, Humphries S, Woo P. The effect of novel polymorphisms in the interleukin-6 (IL-6) gene on IL-6 transcription and plasma IL-6 levels, and an association with systemic-onset juvenile chronic arthritis. J Clin Invest. 1998; 102:1369–76. 10.1172/JCI2629 9769329 PMC508984

[17] Zhou L, Zheng Y, Tian T, Liu K, Wang M, Lin S, Deng Y, Dai C, Xu P, Hao Q, Kang H, Dai Z. Associations of interleukin-6 gene polymorphisms with cancer risk: evidence based on 49,408 cancer cases and 61,790 controls. Gene. 2018; 670:136–47. 10.1016/j.gene.2018.05.104 29842912

[18] Chen L, Zhang Z, Huang J, Jin M. Association between rs1800795 polymorphism in the interleukin-6 gene and the risk of polycystic ovary syndrome: a meta-analysis. Medicine (Baltimore). 2018; 97:e11558. 10.1097/MD.0000000000011558 30024552 PMC6086475

[19] Duan HX, Chen YY, Shi JZ, Ren NN, Li XJ. Association of IL-6 -174G>C (rs1800795) polymorphism with cervical cancer susceptibility. Biosci Rep. 2018; 38:BSR20181071. 10.1042/BSR20181071 30135142 PMC6137247

[20] Liu TZ, Guo ZQ, Wang T, Cao Y, Huang D, Wang XH. Meta-analysis of the role of IL-6 rs1800795 polymorphism in the susceptibility to prostate cancer: evidence based on 17 studies. Medicine (Baltimore). 2017; 96:e6126. 10.1097/MD.0000000000006126 28296724 PMC5369879

